# HPLC and LC–MS/MS-Based Quantitative Characterization of Related Substances Associated with Sotalol Hydrochloride

**DOI:** 10.3390/molecules29030588

**Published:** 2024-01-25

**Authors:** Pengyan Zhu, Xiaojing Shen, Xinting Wang, Xinlan Liu, Yingshuang Zhang, Ke Wang, Wenfen Gao, Xuanjun Wang, Wenjuan Yuan

**Affiliations:** 1 College of Science, Yunnan Agricultural University, Kunming 650201, China; zhupengyan16@163.com (P.Z.);; 2 Key Laboratory of Puer Tea Science, Ministry of Education, Yunnan Agricultural University, Kunming 650201, China; 3 College of Food Science and Technology, Yunnan Agricultural University, Kunming 650201, China; 4 Yunnan Institute for Food and Drug Control, Kunming 650201, China; 5 Yunnan Key Laboratory of Southern Medicinal Resource, School of Chinese Materia Medica, Yunnan University of Chinese Medicine, Kunming 650500, China

**Keywords:** sotalol hydrochloride (STHCl), related substances, characterization, LC–MS/MS, NMR, MTT analysis, acute toxicity

## Abstract

In total, three related substances (RS) associated with sotalol hydrochloride (STHCl) were herein identified with a novel gradient high-performance liquid chromatography (HPLC) protocol. Further characterization of these substances was then performed via liquid chromatography–mass spectroscopy (LC–MS/MS) and nuclear magnetic resonance (NMR) approaches. For these analyses, commercial STHCl samples were used for quantitative HPLC studies and the degradation of STHCl under acidic (1M HCl), alkaline (1M NaOH), oxidative (30% H_2_O_2_), photolytic (4500 Lx), and thermal stress conditions (100 °C) was assessed. This approach revealed this drug to be resistant to acidic, alkaline, and high-temperature conditions, whereas it was susceptible to light and oxidation as confirmed through long-term experiments. The putative mechanisms governing RS formation were also explored, revealing that RS3 was derived from the manufacturing process, whereas RS2 was generated via oxidation and RS1 was generated in response to light exposure. The cytotoxicity of these RS compounds was then assessed using MTT assays and acute toxicity test. Overall, this study provides details regarding the characterization, isolation, quantification, and toxicological evaluation of STHCl and associated RS compounds together with details regarding the precise, specific, and reliable novel HPLC technique, thus providing the requisite information necessary to ensure STHCl purity and safety.

## 1. Introduction

The antihypertensive drug sotalol hydrochloride (STHCl) is used to treat arrhythmias and ischemic heart disease [1,2,3], functioning as a non-selective β-adrenergic antagonist with β-adrenoreceptor blocking activity [4]. First produced in 1966 [5], STHCl was listed in the UK in 1974 and provided with US Food and Drug Administration approval in 1992 [6]. It is now prescribed to treat various ventricular and supraventricular arrhythmias, offering high levels of bioavailability and a long half-life such that it is often used in clinical settings and is now available in over 40 countries worldwide [7]. The toxicological effects of drugs and associated adverse reactions are often attributable to related substances (RSs) derived from these drugs or their degradation products and there is, thus, substantial interest in the development of methods to better assess drug stability and to detect process-related or degradation-related impurities in drug preparations. Chemical synthesis techniques are the most common approach to manufacturing most drugs [5] and the characterization, quantitative analysis, and control of RS content within bulk drug preparations are, thus, critical to effective regulatory assessment efforts.

High-performance liquid chromatography (HPLC) approaches are commonplace when seeking to control for impurities in drug preparations but there have only been a few publications focused on detecting RS associated with STHCl [8,9,10,11]. Analytical methods have been described to separate out known impurities, which include sotalol-related compounds A/B/C and sotalol EP impurity D [5]. However, the extant literature suggests that there have been no thorough efforts to systematically characterize unknown RSs associated with STHCl preparations. Effective characterization and quantitative analysis of these STHCl-derived RS compounds require the establishment of an HPLC method that is accurate and reliable [12].

In this study, an HPLC approach was, thus, developed and used in combination with LC–MS/MS to separate and identify process- and degradation-related substances associated with STHCl [13,14]. Parent ion identification was performed through liquid chromatography–time-of-flight mass spectrometry (LC–TOFMS), while LC–MS/MS was used to characterize fragment ions [15]. In total, three RSs, including two not previously identified, were herein isolated and confirmed through NMR approaches and a review of the literature. The toxicological effects of these RSs on four different cell lines were then tested in an MTT assay [16] and the acute toxicity of RS1 and RS2 was assessed in vivo at a fixed-dose level (200 mg/kg) [17]. The results of these analyses, ultimately, revealed the stability of the developed approach to isolating and characterizing STHCl-associated RSs via HPLC, LC–MS, and NMR approaches [18,19], while also offering in vitro and in vivo evidence regarding the acute toxicity of two newly identified RSs.

## 2. Results and Discussion

### 2.1. HPLC Methodological Standardization and Validation

**Figure 1:**
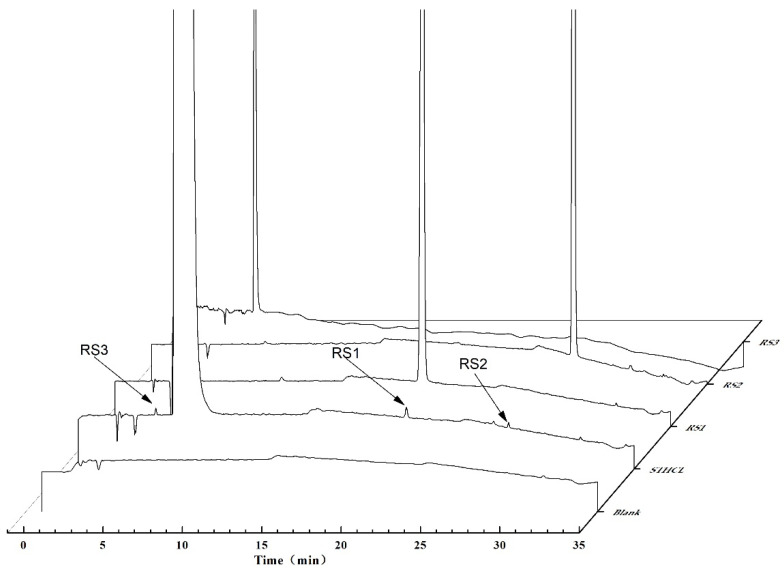
A representative chromatogram highlighting the retention times for the indicated RSs.

System suitability testing for the HPLC system was performed for each validation parameter. As per Section 2.2, these HPLC analyses revealed that three RSs were present in analyzed samples at relative RTs of 19.3, 26.8, and 5.9 min, with a main STHCl peak at 6.1 min. These three substances were, respectively, designated as RS1–3. A representative chromatogram highlighting the retention times for these RSs is presented in Figure 1. The developed HPLC method was next subjected to extensive validation of key assay parameters.

#### 2.1.1. Specificity

Methodological specificity was assessed using test solutions and forced degradation solutions as prepared above, with a DAD detector being employed to evaluate spectral peak purity for all chromatographic peaks. As shown in Table 1 and Figure S1, blank solutions did not exhibit any interference with respect to co-eluting peaks, and a resolution of >3.0 was achieved for all adjacent peaks, consistent with adequate methodological selectivity.

#### 2.1.2. Linearity

The linearity of detector responses for STHCl and RS1–3 was next assessed. The peak area responses for STHCl and these RSs were strictly linear in the 0.5–100 μg/mL concentration range. The corresponding regression formula, regression coefficient, and correction factor values are presented in Table 2 and regression curves are shown in Figure S2.

#### 2.1.3. LOD and LOQ Testing

The respective thresholds used for LOD and LOQ determinations were signal-to-noise ratios of 3:1 and 10:1. The RSD of the areas for six replicate injections at the LOQ concentration was <10% and the respective signal-to-noise ratios at the LOD and LOQ concentrations were less than three and ten. Respective measured LOD values for RS1, RS2, and RS3 were 0.103, 0.0823, and 0.0854 µg/mL, with corresponding LOQ values of 0.309, 0.248, and 0.256 µg/mL. The LOD and LOQ for STHCl were 0.0625 and 0.1875 µg/mL, respectively. The resultant data are presented in Table 3.

#### 2.1.4. Accuracy and Precision

Methodological accuracy and precision were evaluated by injecting multiple levels of STHCl, RS1, RS2, and RS3 standards, with resultant recovery rates ranging from 100.00–116.00% (*w*/*w*). Corresponding percentage recovery values were presented in Table 4 and the RSD was <5%, consistent with good methodological repeatability.

Analyses of intra- and inter-day precision for this method at sample concentrations in the 0.5–100 μg/mL range yielded RSD values of 3.58% or lower for the STHCl, RS1, RS2, and RS3 standards (Table 4). These results, thus, confirmed a high degree of accuracy and precision for this approach.

#### 2.1.5. STHCl Solution Stability

To test the solution stability of STHCl, spiked sample solutions were incubated for 24 h in volumetric flasks that were tightly capped. No significant shifts in peak area were evident in these solution stability tests, confirming that STHCl solutions remained stable for 24 h. When a 1 mg/mL STHCl solution was tested after 24 h, the STHCl, RS1, RS2, and RS3 signals all remained stable (Table 5).

#### 2.1.6. Robustness

STHCl content was assessed under different conditions to ensure this approach was robust and maintained the separation requirements with these changing conditions. Cumulative STHCl RSs in prepared standard solution levels were all <10.0% (Table 6).

### 2.2. Commercial Sample Analyses

The established method was next used to detect the levels of the identified RSs in STHCl bulk drug samples from commercial sources (Samples A–F). The results are presented in Table 7, revealing that RS1 was present at concentrations exceeding 1.0%.

### 2.3. LC–MS and NMR Characterization of STHCl-Associated RSs

**Figure 2:**
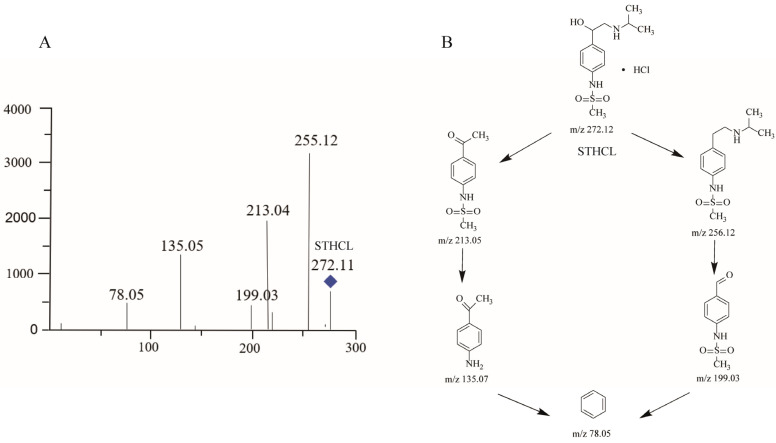
Mass chromatogram and potential fragmentation schemes for STHCl. (**A**) Ionic peaks of STHCl related products obtained from MS/MS studies. (**B**) Cleavage mechanism present in STHCl during storage.

**Figure 3:**
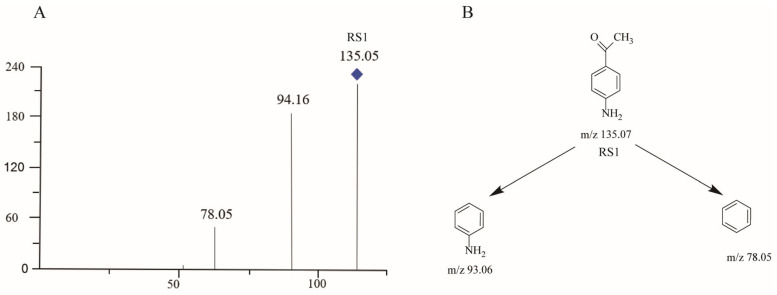
Mass chromatogram and potential fragmentation schemes for RS1. (**A**) Ionic peaks of RS1-related products. (**B**) Possible dissociation mechanisms for the formation of RS1 product ions.

**Figure 4:**
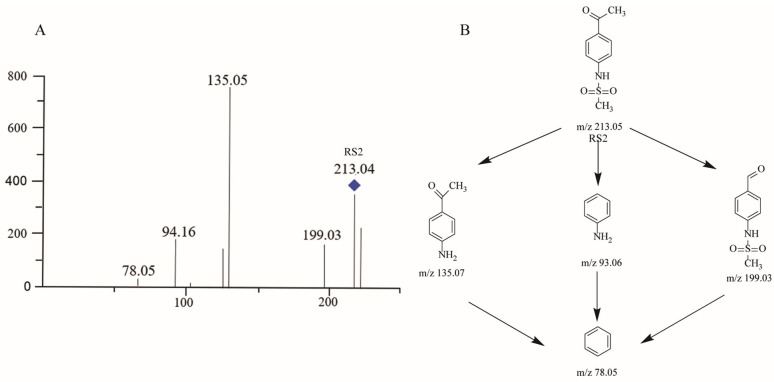
Mass chromatogram and potential fragmentation schemes for RS2. (**A**) Ionic peaks of RS2-related products. (**B**) Possible dissociation mechanisms for the formation of RS2 product ions.

**Figure 5:**
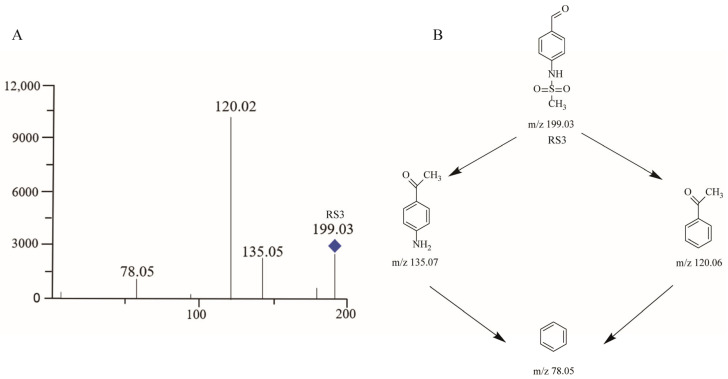
Mass chromatogram and potential fragmentation schemes for RS3. (**A**) Ionic peaks of RS3-related products. (**B**) Possible dissociation mechanisms for the formation of RS3 product ions.

LC–MS and NMR are the most commonly used strategies for the identification of unknown structural information with the best resolved minor components [20]. A typical HPLC chromatogram for STHCl containing the indicated RS impurities is presented in Figure 1. RS1–3 were detected in crude STHCl samples during process development studies and these compounds were then subjected to LC–MS identification, with corresponding mass spectrometric data being shown in Table 8 and total ion chromatograms for these RSs being presented in Figure 2, Figure 3, Figure 4 and Figure 5. The identification of these RSs and their potential fragmentation mechanisms were assessed through LC–MS/MS and RSs were then synthesized to obtain quantities sufficient for NMR analysis. 1H NMR and 13C NMR assignments for RS1 and RS2 are summarized in Table 9 and NMR spectra for RS1-3 are shown in Figure S1. The mass values and RT for all RSs were also confirmed by injecting the isolated RS compounds for HPLC and LC–MS analysis.

#### 2.3.1. STHCl

The mass spectrum for STHCL exhibited a protonated molecular ion [M + H]^+^ at *m*/*z* 273.1268, and MS/MS spectral data revealed five ion peaks at *m*/*z* 255.12, *m*/*z* 213.04, *m*/*z* 199.03, *m*/*z* 135.05, and *m*/*z* 78.05 (Figure 2A). The dissociation mechanisms presented in Figure 2B can explain the formation of these product ions.

#### 2.3.2. RS1

A process-related impurity identified at RT 19.4 min was designated as RS1 and the mass spectrum for RS1 exhibited a protonated molecular ion at *m*/*z* 135.05 (Figure 3 and Figure S4.3). RS1 was a major degradation product generated by oxidizing stress with reduced retention under reverse-phase HPLC conditions. In MS/MS analyses, RS1 yielded two major product ions at *m*/*z* 94.16 and *m*/*z* 78.05 (Figure 3A) and the proposed structure and fragmentation pattern for this RS are presented in Figure 3B. NMR analyses were used to additionally characterize RS1.

The HPLC-based chromatographic isolation of RS1 yielded an amorphous white powder with a quasi-molecular ion [M + H]^+^ at *m*/*z* 136.0759 (calculated for C_8_H_9_NO, 136.0757) in its HRESIMS spectrum, with five magnitudes of unsaturation. Its ^1^H-NMR spectra included one methyl signal [δH 2.47 (H_3_-8)], two amine (NH_2_) liable protons [δH 4.59 (NH_2_)], and two olefinic proton signals (OPS) at δH 7.76 (H-2, 6) and 6.64 (H-3, 5) (Figure S4.1). Its ^13^C-NMR spectra exhibited six carbon resonances that were categorized as two carbonyl resonances at δC 199.2 (C-7), three olefinic bonds at δC 155.4 (C-4), 132.2 (C-2, 6), 126.9 (C-1), and 114.2 (C-3, 5), and one methyl resonance at δC 25.9 (C-8) (Table 9 and Figure S4.2). Based on prior publications, these spectroscopic data supported the identification of RS1 as 4-Aminoacetophenone [21].

#### 2.3.3. RS2

A process-related impurity detected at RT 26.8 min was designated as RS2 and the mass spectrum for RS2 exhibited a protonated molecular ion at *m*/*z* 213.05 (Figure 4A and Figure S5.3). RS2 was the major degradation product generated under thermal stress conditions which resulted in a 5.7-fold increase in RS2 levels. HPLC and LC–MS results suggested that STHCl was degraded into the *m*/*z* 135.05, 94.16, and 78.05 product ions, two of which were the same as those observed for RS1. These product ions may be formed by the mechanisms outlined in Figure 4B. RS2 was further characterized by ^1^H-NMR and ^13^C-NMR approaches (Figures S5.1 and S5.2). RS2 was isolated as an amorphous white powder with a quasi-molecular ion [M + H]^+^ at *m*/*z* 212.0386 (calculated for C_9_H_11_NO_3_S, 212.0387) in its HRESIMS spectrum, with seven magnitudes of unsaturation. Its ^1^H-NMR spectrum included two methyl signals [δH 2.57 (H_3_-8), 3.06 {H_3_-[S(=O)_2−_]}], one amine (NH) liable proton [δH 4.59 (H-N)], and two OPS at δH 7.32 (H-2, 6) and 8.00 (H-3, 5). Its ^13^C-NMR spectrum exhibited seven carbon resonances that were categorized as two carbonyl resonances at δC 199.4 (C-7), olefinic bonds at δC 144.7 (C-4), 133.6 (C-2,6), 131.3 (C-1), and 119.0 (C-3, 5), and two methyl resonances at δC39.9 (Me-[S(=O)_2−_]) and 19.3 (C-8). These spectroscopic data led to the identification of RS2 as N-(4-Acetylphenyl) methanesulfonamide [22].

#### 2.3.4. RS3

LC–MS analyses in ESI mode revealed a process-related impurity at RT 5.9 min that was designated as RS3. High-resolution TOF data suggested that the molecular formula for RS3 may be C_8_H_9_NO_3_S (Figure 5A and Figure S6) and RS3 has previously been reported as sotalol-related compound B. The protonated molecule was evident at *m*/*z* 198.0231 and it fragmented to yield the *m*/*z* 135.05, *m*/*z* 120.02, and *m*/*z* 78.05 product ions through the fragmentation pattern shown in Figure 5B.

### 2.4. Forced Degradation and Long-Term Storage Analyses

**Figure 6:**
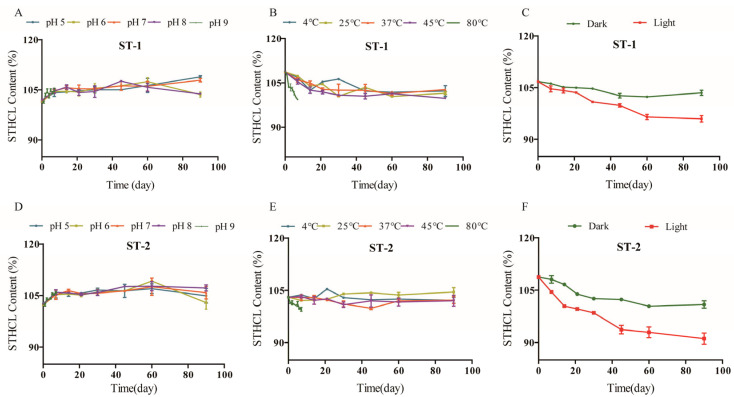
Two batches of STHCl API (nos. ST-1 and ST-2) content under different conditions. (**A**) shows the storage content of ST-1 at different pH; (**B**) shows the storage content of ST-1 at different temperatures; and (**C**) shows the storage content of ST-1 under light. (**D**–**F**) show the storage content of ST-2 at different pH, temperature, and light.

Stress testing efforts can be used to gauge the intrinsic stability of a given drug based on the establishment of the associated degradation pathways, thereby enabling the identification of likely degradation products [23]. These findings can inform manufacturing processes, drug storage, and the determination of an appropriate expiration date. When prepared STHCl samples were exposed to acidic, alkaline, or high-temperature stress conditions, no major degradation impurities were detected. Under conditions of strong light exposure, a minor degradation impurity (RS2) was detected at RT 26.8 min, with the peak area for RS2 under these conditions being 7.8-fold larger than the RS2 peak area for the prodrug. Under oxidizing conditions, a 5.7-fold increase in peak area for RS1 was observed. These results, thus, confirmed the sensitivity of STHCl to light- and oxidation-induced degradation (Table 1). Peak purity analyses with a PDA detector confirmed the homogeneity of the STHCl peak in all stress testing samples, with mass balance results in the 99.3–102.5% range. STHCl remained stable when stored for 90 days in a long-term storage assay at different temperatures and pH conditions, with light exposure. STHCl content in both batches gradually declined throughout storage (Figure 6), with the STHCl content in one batch being 90.11% following the 90-day incubation. As RS1 and RS2 are synthetic components of STHCl, which appears to be less stable when exposed to bright light and oxidizing conditions, such decomposition may have occurred over the course of storage, emphasizing the need to avoid light and oxidation during the storage of this drug.

### 2.5. Cytotoxicity and Acute Toxicity Analyses

**Figure 7:**
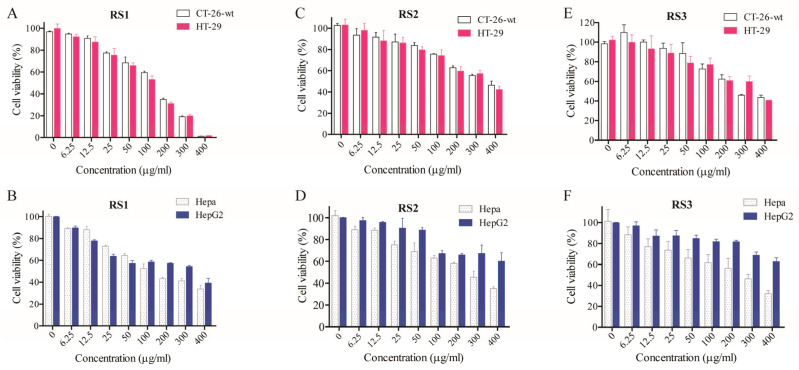
Quantification of the inhibitory effects of RS treatment for 24 h on the indicated cancer cell lines. (**A**,**B**) shows the inhibition of four cancer cells by RS1; (**C**,**D**) describes the inhibition of four cells by RS2; (**E**,**F**) shows the inhibition of four cells by RS3.

In vitro analyses were next used to better understand the toxicological and biological characteristics of RS1 and RS2. The cytotoxicity of these two RSs was assessed by using them to treat the CT26.WT, HT-29, HepG-2, and HePa 1-6 cancer cell lines (Figure 7, Table 10), with DMSO as a control [24]. The respective IC_50_ values for RS1 when used to treat CT26.WT and HT-29 cells were 47.44 and 64.81 μg/mL.

To gain additional insight into the safety of these RSs, Kunming mice were used to conduct acute toxicity studies. When these mice were dosed with RS1 or RS2 at 200 mg/kg, no evidence of death or other abnormalities was observed, thus suggesting that these compounds do not have any bearing on the safety of STHCl when used at the prescribed dose.

## 3. Experimental Methods

### 3.1. Chemicals and Reagents

All chemicals used in this study were of analytical grade, while HPLC-grade solvents were used for all analyses. HPLC-grade acetonitrile was from the MREDA Company Inc. (Beijing, China). Analytical-grade ammonium acetate, formic acid, sodium hydroxide, hydrogen peroxide, and hydrochloric acid were from the Tianjin Damao Chemical Reagent Factory (Tianjin, China). Ultrapure water was from the Wahaha Limited Group Co. (Hangzhou, China). STHCl was from the Yunnan Provincial Institute of Food and Drug Control (batch nos. ZY190402, ZY190501, ZY190502, 101190607, 101190608, and 101190609). Reference STHCl was obtained from the Chinese Food and Drug Inspection Institute (batch no. 100737-200501). The references RS compounds 4-(Methylsulfonamido) denzaldehyde (Sotalol Related Compound B (batch no. R001R0 USP, ≥98.0% pure) and 4-Aminoacetophenone (batch no. A800871, ≥99.0% pure) were from the Shanghai Macklin Biochemical Technology Co., Ltd. (Shanghai, China), while N-(4-Acetylphenyl) methanesulfonamide (batch no. F700955, ≥98.0% pure) was from the Shanghai Yuanye Bio-Technology Co., Ltd. (Shanghai, China). HepG-2, HePa 1-6, HT-29, and CT26.WT cells were obtained from the Kunming Wildlife Cell Bank of the Chinese Academy of Sciences. RPMI-1640, high-glucose DMEM, and fetal bovine serum (FBS) were obtained from Gibco (New York, NY, USA) and 0.25% trypsin-EDTA and penicillin-streptomycin were from Solarbio (Beijing, China).

### 3.2. HPLC

An Agilent 1260 HPLC system with a DAD detector was used to conduct HPLC analyses using the Thermo Acclaim RP-C18 column (250 × 4.6 mm, 5 µm) (Waltham, MA, USA). For separation, the mobile phase consisted of aqueous 5 mM ammonium acetate with 0.02% formic acid (A) and acetonitrile (B). Using a flow rate of 1.0 mL/min, the following settings were used for gradient elution: 0–20.0 min, 0–20% B; 20.0–30.0 min, 20–55% B; and 30.0–35.0 min, 55% B. A 20 µL injection volume was used and the column was maintained at 30 °C, with 228 nm as the wavelength for detection.

#### 3.2.1. Sample and Standard Preparation

Samples were prepared from bulk STHCl samples (6 batches, *n* = 3), which were added to a 1:4 acetonitrile/water solution and diluted to 100 μg/mL. STHCl standards were prepared by weighing an appropriate amount of STHCl standard and suspending in a 1:4 acetonitrile/water solution and stepwise dilution to 50, 10, 5, 1, and 0.5 μg/mL. RS1, RS2, and RS3 standard solutions were prepared using the same approach at concentrations of 50, 10, 5, 1, and 0.5 μg/mL.

#### 3.2.2. Methodological Validation

Per the Chinese Pharmacopoeia 2020, Part IV, analytical methods were validated for pharmaceutical quality, including analyses of specificity, sensitivity, linearity, range, accuracy, precision, stability, and robustness [25].

##### Specificity

Methodological specificity was assessed through analyses of STHCl samples that had been subjected to acidic, alkaline, photolytic, oxidative, or thermolytic degradation [26]. Potential degradation product interference in stress-degraded samples at the STHCl retention time and the retention times for RSs were assessed.

##### Sensitivity

Limit of detection (LOD) and limit of quantitation (LOQ) preparations were used to assess methodological sensitivity, calculating the LOD and LOQ based on respective signal-to-noise (S/N) ratios of 3:1 and 10:1. The LOD and LOQ values were confirmed via the injection of six samples at the LOD and LOW limits for each analyte and the peak area % RSD was limited to 33% and 10% of STHCl for LOD and LOQ, respectively, as per ICH guidelines [27].

##### Linearity and Range

STHCl standard solutions were prepared at various concentrations (0.5, 1, 5, 10, 50, and 100 μg/mL). Calibration curves were established by plotting peak area ratios against different STHCl standard and RS concentrations. Least-squares regression analyses were used to assess linearity and curves were not required to intersect with the origin. The LOQ was identified as the lowest concentration on this standard curve.

##### Accuracy and Precision

Accuracy and precision analyses were performed using STHCl, RS1, RS2, and RS3 standard solutions prepared at various concentrations (10, 50, and 100 μg/mL). Repeatability analyses were performed by assessing five sample replicates in one day for intra-day tests and on three consecutive days for inter-day tests. Accuracy was defined as the percentage of recovery in these analyses, whereas precision was determined with the relative standard deviation (% RSD).

##### Stability

For stability testing, three STHCl standard concentrations (10, 50, and 100 μg/mL) were stored at room temperature, assessing the peak areas for each sample at six time points using the established chromatographic conditions.

##### Robustness

Methodological robustness was assessed by evaluating whether or not results were impacted by small shifts in assay conditions in order to better provide a foundation for the use of these methods in a routine testing context. Variations in experimental conditions were as follows: detection wavelength (228 ± 5 nm), column temperature (30 ± 2 °C), and flow rate (1.0 ± 0.2 mL/min), with different instruments (Agilent 1260 and Shimadzu 2010) also being employed. The composition of mobile phase A was also adjusted to ammonium acetate concentrations of 4 or 6 mM. STHCl peak retention times were assessed when using these various conditions.

### 3.3. LC–MS/MS

HPLC conditions were employed for HPLC-UV and LC-MS detection. The RS parent ions were determined based on LC-TOFMS analyses performed with an Agilent 1290 series HPLC system and an Agilent 6540 TOFMS instrument with an ESI source using the following parameters: spray voltage = 3500 V; capillary temperature = 300 °C; gas pressure = 30 psi; and aux gas pressure = 30 psi. The MS was operated in the full-scan mode with an *m*/*z* range of 50~1700 in positive/negative mode.

Fragment ion identification was performed with an Agilent UPLC/Q-TOF liquid mass spectrometer (Agilent, Santa Clara, CA, USA) composed of a quaternary pump solvent management system, with an autosampler and an online degasser, using the Thermo TSQ Quantum MS instrument as an ESI source with the following source parameters: spray voltage = 3500 V; capillary temperature = 300 °C; gas pressure = 30 psi; ion sweep gas pressure = 1.0 psi; tube lens offset = 135 V; skimmer offset = 65 V; and aux gas pressure = 30 psi. Product ion scan mode was used to operate the MS instrument.

### 3.4. Isolation and Identification

Products were purified via preparative HPLC (Agilent-1260) using an Agilent XDB-C18 (5 µm, 9.4 × 250 mm) column, with a mobile phase composed of aqueous 5 mM ammonium acetate with 0.02% formic acid and acetonitrile (80:20, *v*/*v*). The flow rate was 2.5 mL/min, and the detection wavelength was 228 nm. The RS1, RS2, and RS3 fractions were lyophilized two times and HPLC confirmed the purity of these products (98.5%) (Figure S1). Isolated impurities were identified through comparisons of spectroscopic and physical findings (^1^H-NMR, ^13^C-NMR, and MS) with prior publications.

### 3.5. Forced Degradation and Long-Term Studies

Forced degradation solutions were prepared by subjecting STHCl to a range of stress conditions in line with the International Conference on Harmonization (ICH) guideline Q1A(R2) [28]. Briefly, STHCl solutions were subjected to hydrolytic (acidic, alkaline, and neutral), thermal, photolytic, and oxidative degradation [29], as follows: acidic hydrolysis (1 M HCl, 37 °C, 30 min), alkaline hydrolysis (1 M NaOH, 37 °C, 4 min), oxidation (30% H_2_O_2_, 37 °C, 30 min), thermal degradation in a heated water bath (H_2_O, 100 °C, 30 min), photolytic degradation (4500 Lx, 16 d), and neutralizing the acidic and alkaline samples prior to dilution [30]. The dilution solvents detailed above were used to dilute samples for final STHCl concentrations of 1 mg/mL and samples were filtered prior to further analysis.

For long-term experiments, STHCl was stored under conditions designed to mimic the actual conditions under which this drug is stored in order to guide the establishment of appropriate drug expiration dates. Two test material batches were prepared and the impacts of temperature (4 °C, 25 °C, 37 °C, 45 °C, and 80 °C) on stability were assessed at a pH of 6.0 while protected from light and air, assessing STHCl content after 0, 7, 14, 21, 30, 45, 60, and 90 days for all samples other than those stored at 80 °C, which were analyzed daily from 0–7 days. The effects of different pH levels (5.0, 6.0, 7.0, 8.0, and 9.0) on stability were assessed at 25 °C while protected from light and air, adjusting the pH with 1 M NaOH or 1 M HCl. Levels of STHCl were assessed after 0, 7, 14, 21, 30, 45, 60, and 90 days for all samples other than those stored at a pH of 9.0, which were analyzed daily from 0–7 days. For analyses of photodegradation, STHCl was stored at 25 °C under closed conditions and one sample was protected from light whereas the other was exposed to a light intensity of 2000 lux, analyzing STHCl content on days 0, 7, 14, 21, 30, 45, 60, and 90. Three replicates were used for all treatments and samples were collected prior to and following storage under these conditions for liquid phase analysis.

### 3.6. Cytotoxicity Assays

To test in vitro cytotoxicity, the CT26.WT and HT-29 colorectal cancer and the HepG-2 and HePa 1–6 liver cancer cell lines were cultured in DMEM or RPMI-1640 containing 10% FBS in a humidified 37 °C 5% CO_2_ incubator. An MTT assay was used to assess cell survival as reported previously [31,32]. Briefly, 200 μL of cells suspended at 1 × 10^4^ cells/mL were added to individual wells in a 96-well plate and allowed to attach for 8–16 h, followed by the addition of the test compounds at a range of concentrations and incubation for 24 h, using DMSO (Sigma, Louis, MO, USA) as a negative control. Conditions were replicated five times. Then, 20 μL of MTT (5 mg/mL) was added per well, followed by a further 4 h incubation at 37 °C. Media were then removed and replaced with 150 μL of DMSO, followed by incubation for 10 min at room temperature with shaking. Absorbance at 492 nm was then assessed and blank controls were used to eliminate the background signal. Viability was analyzed as a percentage of control cells [33,34].

### 3.7. Acute Toxicity Assays

The acute toxicity of RS1 and RS2 was assessed as a means of assessing the safety of these compounds in order to inform safe clinical drug use. In total, 18 Kunming mice (9 male, 9 female) were randomized into 3 groups according to sex and body weight, and they were orally administered RS1 and RS2 by gavage at a dose of 200 mg/kg, while control mice were administered an equal volume of saline. Side effects and death in these mice were recorded over 24 h [35,36].

### 3.8. Statistical Analysis

The experimental data were analyzed using one-way ANOVAs by SPSS19.0 (IBM, Armonk, NY, USA) and GraphPad Prism 5.0 (Graph-Pad Software, Inc., La Jolla, CA, USA). Graphs were created in Origin 8.5 (Origin Institute Inc., Victoria, TX, USA). All data were expressed as mean ± SD, *p* < 0.05 and differences were considered statistically significant. The RSD value reflects the precision of the analytical results in the assay.

## 4. Conclusions

In summary, a reliable HPLC approach was herein developed for the quantitative analysis of STHCl-associated RSs based on ICH guidelines regarding stress conditions. The three identified compounds (RS1–3) were isolated and subjected to extensive NMR (^1^H, ^13^C, and DEPT) and LC-MS/MS characterization. The predicted mechanisms likely responsible for the formation of these RSs were also discussed. These substances were derived from manufacturing processes and forced degradation conditions, with RS1 being the major degradation product produced under oxidizing conditions, while RS2 was the major degradation product generated under strong light exposure and RS3 was derived from the manufacturing process. The ability of RS1 and RS2 to inhibit the growth of cancer cell lines was also assessed, revealing that RS1 exhibited moderate cytotoxicity when used to treat the CT26.WT and HT-29 cell lines, with respective IC_50_ values of 47.44 and 64.81 ug/mL. Based on acute toxicity testing performed in mice, STHCl was confirmed to be safe based on the lack of any apparent toxic effects from RS1 or RS2 treatment. RS-focused research is vital to ensuring the quality, security, and rigorous assessment of pharmacological products. The present results emphasize the importance of maintaining appropriate drug storage conditions to avoid any adverse outcomes, providing valuable guidance for quality control efforts in the context of STHCl manufacturing.

## Figures and Tables

**Table 1:** Forced degradation study parameters.

Samples	STHCL (A)	RS1 (A)	RS2 (A)	RS3 (A)	Resolution between STHCl Peak and Nearest Peak	Mass Balance (%)	Peak Purity of STHCl
Control	2.37 × 10^7^	1.5 × 10^5^	9.15 × 10^4^	1.03 × 10^5^	27.86	99.9979	0.966
Acidic hydrolysis	2.36 × 10^7^	1.3 × 10^5^	2.26 × 10^4^	1.24 × 10^5^	24.73	99.2996	0.959
Alkaline hydrolysis	2.35 × 10^7^	1.26 × 10^5^	8.98 × 10^4^	1.16 × 10^5^	29.95	99.1133	0.989
Thermal degradation	2.44 × 10^7^	1.12 × 10^5^	1.16 × 10^4^	1.12 × 10^5^	18.96	102.4562	0.99
Oxidation degradation	2.31 × 10^7^	8.55 × 10^5^	9.54 × 10^4^	1.13 × 10^5^	29.78	100.4924	0.971
Light degradation	2.26 × 10^7^	4.05 × 10^5^	7.14 × 10^5^	1.33 × 10^5^	28.02	99.1973	0.981

**Table 2:** Linearity data.

Analyte	Calibration Equation	Concentration Range	Correlation Coefficient (r^2^)
STHCl	y = 10.553x + 1.9967	0.5~100 μg/mL	r = 0.9999
RS1	y = 26.443x + 65.468		r = 0.9994
RS2	y = 2.8121x − 0.0571		r = 1.0000
RS3	y = 1.7131x + 1.7042		r = 0.9999

**Table 3:** LOD, LOQ, and response factor data.

Compound	LOD	LOQ	S/N	
	μg/mL	μg/mL	LOD Limit: ≥3	LOQ Limit: ≥10
STHCL	0.0625	0.1875	10	15
RS1	0.103	0.309	8	15
RS2	0.0823	0.248	6	14
RS3	0.0854	0.256	7	16

**Table 4:** RS recovery and precision data.

Compound	C (μg/mL)	Accuracy		Interday Precision (*n* = 5)		Intraday Precision (*n* = 5)	
				Precision		Precision	
		Recovery Rate (%)	RSD (%)	A	RSD (%)	A	RSD (%)
STHCL	10	104.23 ± 3.28	3.15	101.075 ± 0.05	0.05	101.11 ± 0.59	2.26
	50	106.32 ± 1.85	1.74	529.328 ± 4.08	0.77	529.53 ± 10.43	1.97
	100	108.23 ± 3.98	3.68	1069.49 ± 24.06	2.25	1069.54 ± 13.58	1.27
RS1	10	105.17 ± 4.53	4.31	254.1 ± 9.10	3.58	254.39 ± 3.21	1.26
	50	116 ± 2.84	2.45	1320.13 ± 6.07	0.46	1321.87 ± 9.12	0.69
	100	104.33 ± 3.67	3.52	2652.95 ± 8.49	0.32	2653.12 ± 12.73	0.48
RS2	10	115.17 ± 4.38	3.8	289.34 ± 3.65	1.26	289.28 ± 5.61	1.94
	50	107.5 ± 2.74	2.55	1404.11 ± 9.69	0.69	1403.88 ± 11.88	0.92
	100	105.6 ± 4.34	4.11	2821.58 ± 13.54	0.48	2822.8 ± 9.32	0.33
RS3	10	102 ± 1.67	1.64	169.08 ± 2.77	1.64	169.1 ± 5.79	1.85
	50	112.5 ± 1.76	1.56	850.3 ± 8.69	1.35	850.5 ± 13.78	1.62
	100	104.33 ± 2.86	2.75	1701.05 ± 34.36	2.02	1700.85 ± 20.92	1.23

**Table 5:** RS stability.

Compound	C (μg/mL)	Time (h)						RSD (%)
		0	2	4	6	12	24	
STHCL	10	103.25	102.73	101.47	100.99	103.46	100.49	1.22
	50	528.42	527.51	534.25	533.33	521.57	525.61	0.90
	100	1054.2	1066.49	1069.97	1073.12	1079.05	1076.65	0.83
RS1	10	570.2	548.18	559.15	555.21	553.95	544.24	1.63
	50	1322.39	1321.15	1343.95	1309.85	1315.31	1322.5	0.88
	100	2701.3	2688.95	2700.9	2765.9	2699.75	2679.4	1.13
RS2	10	569.2	534.18	565.15	553.21	554.95	544.24	2.35
	50	1393.65	1400.75	1399.63	1398.85	1413.71	1413.97	0.60
	100	2798.95	2813.8	2822.5	2870.92	2813.1	2812.9	0.89
RS3	10	159.398	165.329	173.396	176.413	177.42	169.41	4.09
	50	839.39	845.15	857.3	860.14	850.4	853.46	0.91
	100	1691.95	1700.45	1699.24	1688.37	1702.85	1719.63	0.64

**Table 6:** Methodological robustness results.

Condition		t_R_ (min)	RSD of Concentration (%)
Detection wavelength (nm)	233	6.01	0.37
	228	6.07	
	223	6.73	
Column temperature (°C)	32	5.97	1.21
	30	6.134	
	28	9.01	
Flow rate (mL/min)	1.2	5.04	0.76
	1	6.35	
	0.8	9.21	
Instrument	Agilent 1260	6.34	4.21
	Shimadzu 2010	10.89	
Mobile Phase A (aqueous ammonium)	4 mM	8.13	0.56
	5 mM	6.88	
	6 mM	5.21	

**Table 7:** Impurity measurements for different STHCl drug samples (A–F).

Sample	Content (μg/mL)		
	RS1	RS2	RS3
Sample A 101190607	1.532	0.861	0.36
Sample B 101190608	1.424	0.798	0.31
Sample C 101190609	1.591	0.823	0.33
Sample D ZY190402	1.523	0.99	0.71
Sample E ZY190501	1.493	0.901	0.98
Sample F ZY190502	1.408	0.869	0.85

**Table 8:** STHCl mass spectra result for STHCl and associated RSs.

Name of Related Substances	t_R_ (min)	Observed Ion Mass (*m*/*z*)	Theoretical Ion Mass (*m*/*z*)	Proposed Molecular Formula [M + H]^+^\[M − H]^−^	Error (ppm)	Product Ions
STHCl	6.074	273.1268	273.1267	C_12_H_20_N_2_O_3_S	−0.37	255.12, 213.04, 199.03, 135.05, 78.05
RS1	19.351	136.0759	136.0757	C_8_H_9_NO	−1.47	135.05, 94.16, 78.05
RS2	26.846	212.0386	212.0387	C_9_H_11_NO_3_S	0.47	199.03, 135.05, 94.16, 78.05
RS3	5.921	199.0231	199.0230	C_8_H_9_NO_3_S	−0.50	135.05, 120.02, 78.05

**Table 9:** Relative NMR assignments for RS1 and RS2.

	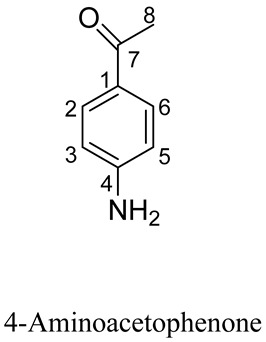		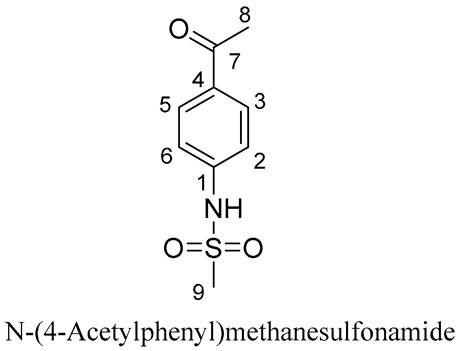	
Position	δ ppm (CD_3_OD, 500 MHZ)		δ ppm (CD_3_OD, 500 MHZ)	
	^1^H	^13^C (DEPT)	^1^H	^13^C(DEPT)
1	-	126.8 (C)	-	144.6 (C)
2, 6	7.74–7.77 (m, 2H)	132.1 (CH)	7.30–7.33 (m, 2H)	118.9 (CH)
3, 5	6.62–6.65 (m, 2H)	114.1 (CH)	7.97–8.00 (m, 2H)	131.3 (CH)
4	-	155.4 (C)	-	133.5 (C)
7	-	199.2 (C)	-	199.9 (C)
8	2.47 (s, 3H)	25.8 (CH_3_)	2.57 (s, 3H)	26.4 (CH_3_)
9	-	-	3.06 (s, 3H)	39.8 (CH_3_)
-NH_n_	4.59 (s, 1H)	-	4.59 (s, 1H)	-

**Table 10:** IC_50_ Values (μg/mL) for RS1, RS2, and RS3 for tumor cell lines.

Name	Hepa	HepG-2	CT26.wt	HT-29
RS1	144.33	237.67	47.44	64.81
RS2	197.7	612.03	456.16	371.38
RS3	207.75	808.75	224.14	241.92

## Data Availability

Data are contained within the article and Supplementary Materials.

## Supplementary Materials

The following supporting information can be downloaded at: https://www.mdpi.com/article/10.3390/molecules29030588/s1, Figures S1–S6.3: Purity of RS1, RS2 and RS3 (Figure S1), Linear relationships of STHCL, RS1, RS2, RS3 (Figure S2), HRESIMS of STHCL, RS1, RS2, RS3 (Figures S3, S4.3, S5.3 and S6.3), ^1^H NMR, and ^13^C NMR of RS1, RS2, and RS3 (Figures S3, S4.1, S4.2, S5.1, S5.2, S6.1 and S6.2).
