# Supplementary material for: HPLC and LC–MS/MS-Based Quantitative Characterization of Related Substances Associated with Sotalol Hydrochloride
Source: Molecules. 2024 Jan 25;29(3):588. doi: 10.3390/molecules29030588 (PMC10856746; doi:10.3390/molecules29030588)
Supplement: Supplementary file 1 [file molecules-29-00588-s001.zip › molecules-2833946-supplementary.pdf]

HPLC and LC–MS/MS-Based Quantitative Characterization of Related Substances Associated with Sotalol Hydrochloride

Pengyan Zhu [1,2,†], Xiaojing Shen [1,2,†], Xinting Wang [2,3], Xinlan Liu [2,3], Yingshuang Zhang [1], Ke Wang [1], Wenfen Gao [4], Xuanjun Wang [5,*] and Wenjuan Yuan [1,2,*]

[1] College of Science, Yunnan Agricultural University, Kunming 650201, China; zhupengyan16@163.com (P.Z.); 2013017@ynau.edu.cn (X.S.); 15559792790@163.com (Y.Z.); 13118788175@163.com (K.W.)

[2] Key Laboratory of Puer Tea Science, Ministry of Education, Yunnan Agricultural University, Kunming 650201, China; 18787628093@163.com (X.W.); liuxinlan15@163.com (X.L.)

[3] College of Food Science and Technology, Yunnan Agricultural University, Kunming 650201, China

[4] Yunnan Institute for Food and Drug Control, Kunming 650201, China; 19187160596@163.com

[5] Yunnan Key Laboratory of Southern Medicinal Resource, School of Chinese Materia Medica, Yunnan University of Chinese Medicine, Kunming 650500, China

* Correspondence: jwang@ynau.edu.cn (X.W.); yuanwj0805@126.com (W.Y.); Tel./Fax: +86-871-6522-6191 (W.Y.)

† These authors contributed equally to this work.

Supporting information

Figure S1 The purity of **RS1**, **RS2** and **RS3** was detected by HPLC
Figure S2 Linear relationships of **STHCL**, **RS1**, **RS2**, **RS3**
Figure S3 HRESIMS spectrum of **STHCL**
Figure S4.1 ^{1}H NMR spectrum of **RS1** in CD_3OD
Figure S4.2 ^{13}C NMR and dept spectrum of **RS1** in CD_3OD
Figure S4.3 HRESIMS spectrum of **RS1**
Figure S5.1 ^{1}H NMR spectrum of **RS2 in** CD_3OD
Figure S5.2 ^{13}C NMR and dept spectrum of **RS2** in CD_3OD
Figure S5.3 HRESIMS spectrum of **RS2**
Figure S6.1 ^{1}H NMR spectrum of **RS3** in $CDCl_3$
Figure S6.2 ^{13}C NMR and dept spectrum of **RS3** in CDCl3
Figure S6.3 HRESIMS spectrum of **RS3**

Figure S1. The purity of RS1, RS2 and RS3 was detected by HPLC

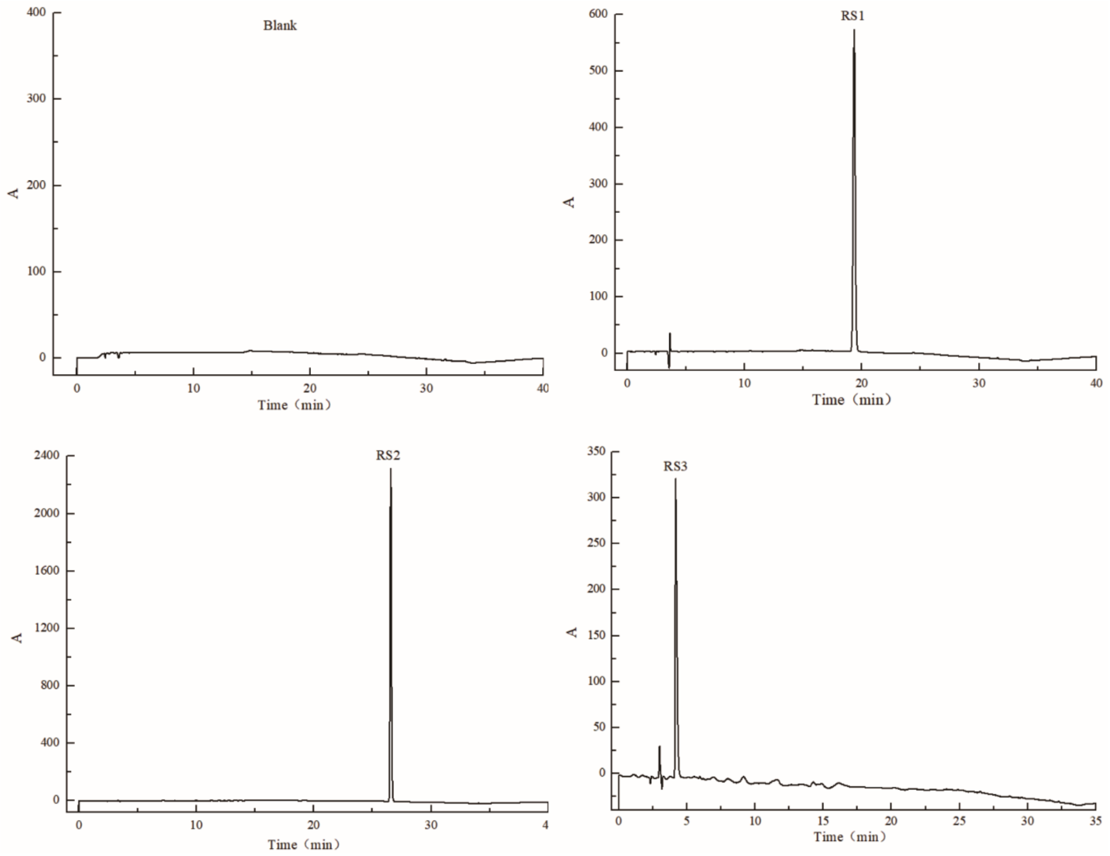

Figure S2. Linear relationships of STHCL, RS1, RS2, RS3

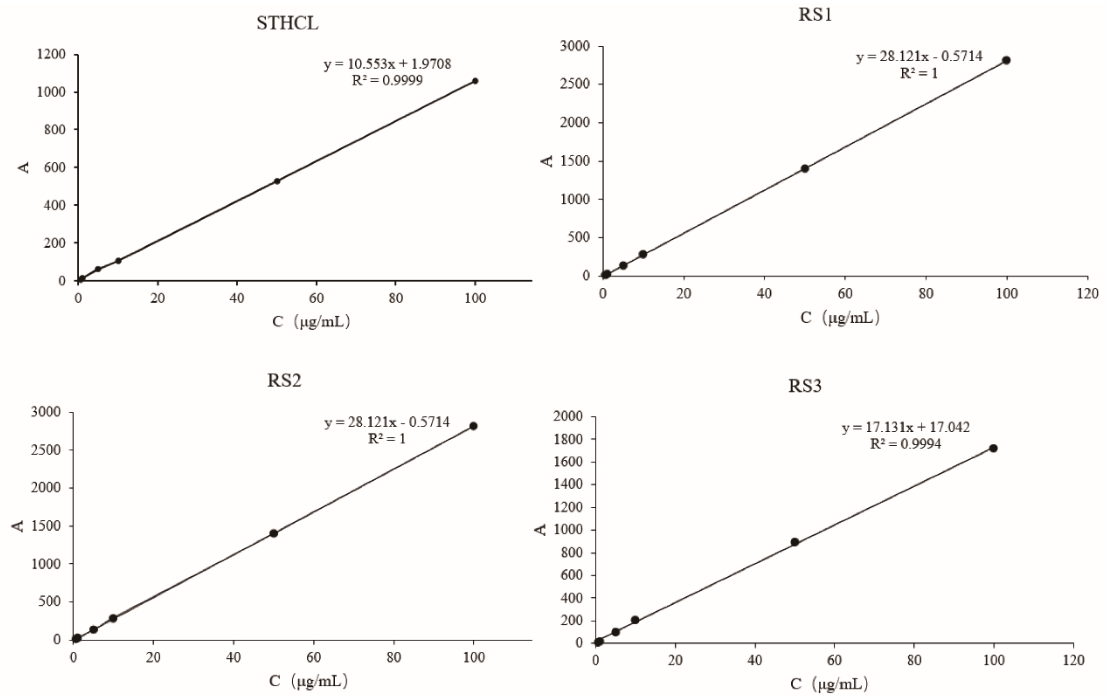

Figure S3 HRESIMS spectrum of STHCL

Qualitative Analysis Report

**Data Filename**	STHCL.d	**Sample Name**	STHCL
**Sample Type**	Sample	**Position**	P1-B1
**Instrument Name**	Instrument 1	**User Name**	
**Acq Method**	s.m	**Acquired Time**	8/22/2022 4:42:21 PM
**IRM Calibration Status**	Success	**DA Method**	PCDL.m
**Comment**			

**Sample Group**		**Info.**
**Acquisition SW**	6200 series TOF/6500 series	
**Version**	Q-TOF B.05.01 (B5125.2)	

User Spectra

Fragmentor Voltage	Collision Energy	Ionization Mode
135	0	ESI

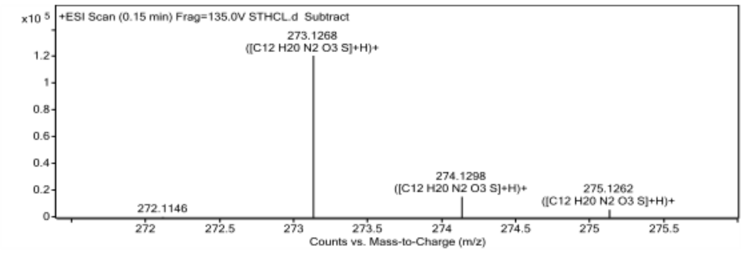

Peak List

m/z	z	Abund	Formula	Ion
255.1161	1	51613.22		
256.1186	1	5509.75		
271.1111	1	8149.53		
273.1268	1	120986.8	C12 H20 N2 O3 S	(M+H)+
274.1298	1	15877.07	C12 H20 N2 O3 S	(M+H)+
275.1262	1	6431.93	C12 H20 N2 O3 S	(M+H)+
701.4951	1	18975.58		
702.4977	1	6812.53		
814.5796	1	13586.98		
815.5827	1	6741.11		

Formula Calculator Element Limits

Element	Min	Max
C	3	60
H	0	120
O	0	30
N	0	3
S	0	3

Formula Calculator Results

Formula	CalculatedMass	CalculatedMz	Mz	Diff. (mDa)	Diff. (ppm)	DBE
C12 H20 N2 O3 S	272.1195	273.1267	273.1268	-0.10	-0.37	4.0000

--- End Of Report ---

Figure S4.1 ^{1}H NMR spectrum of RS1 in CD_3OD

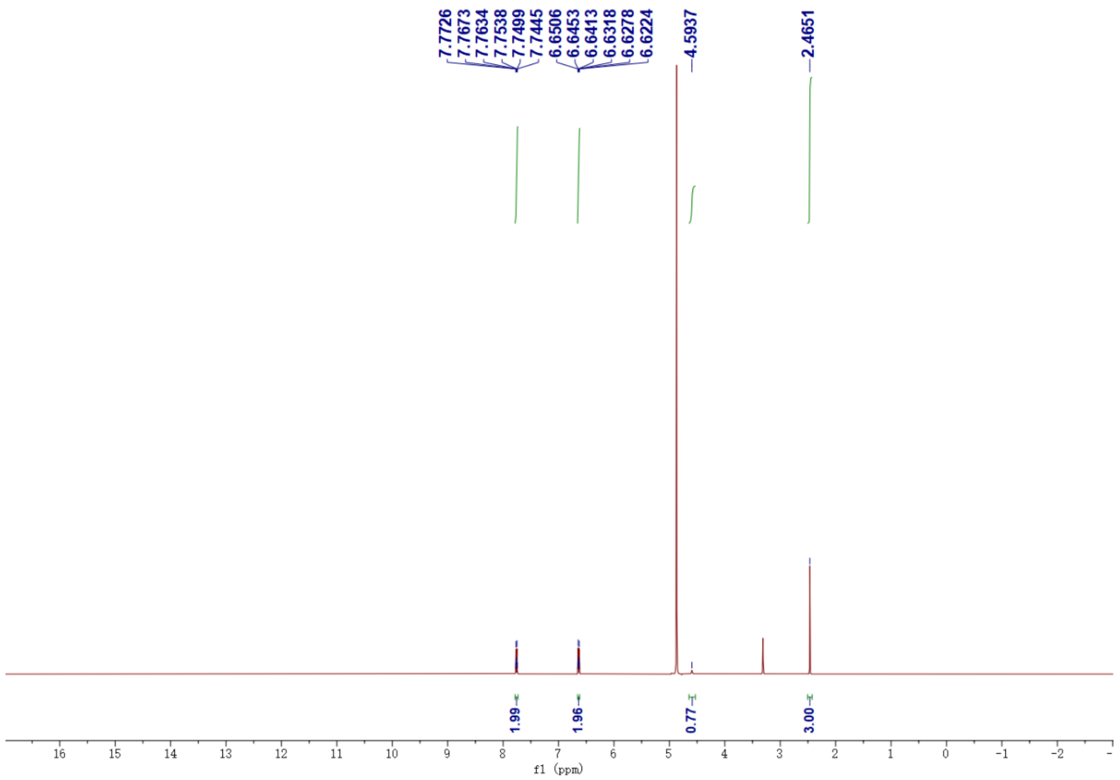

Figure S4.2 ^{13}C NMR spectrum of RS1in CD_3OD

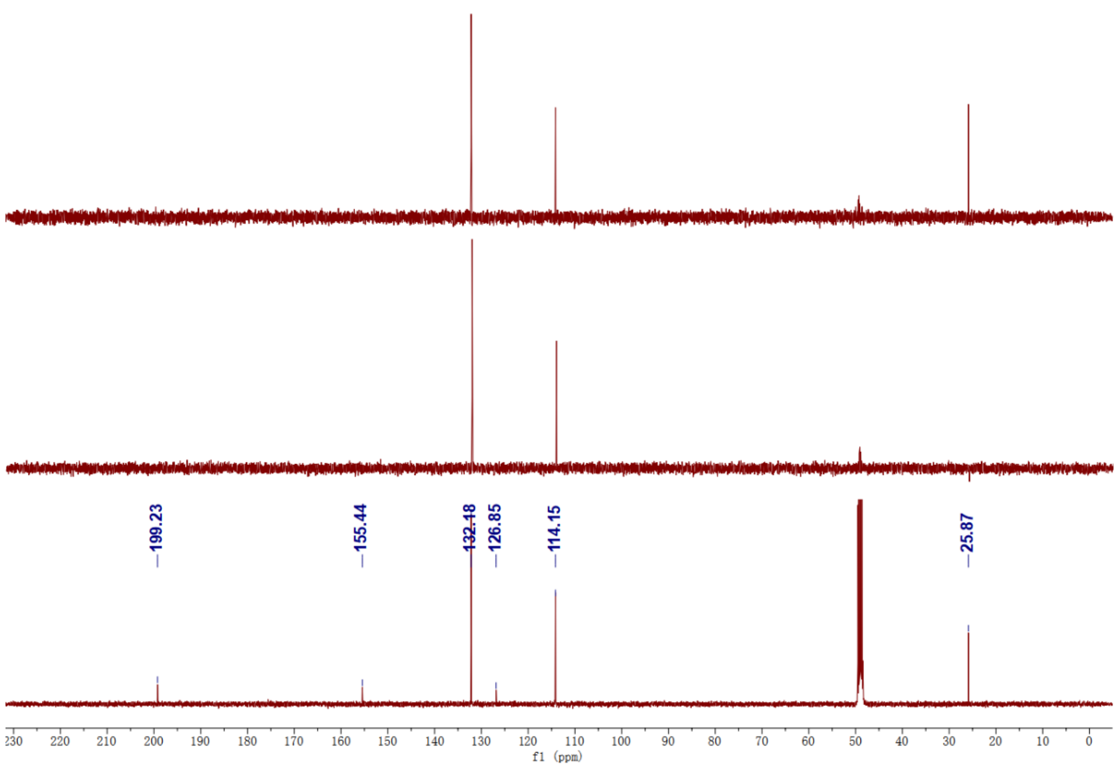

Figure S4.3 HRESIMS spectrum of RS1

Qualitative Analysis Report

**Data Filename**	RS1.d	**Sample Name**	RS1
**Sample Type**	Sample	**Position**	P1-B2
**Instrument Name**	Instrument 1	**User Name**	
**Acq Method**	s.m	**Acquired Time**	8/22/2022 4:43:32 PM
**IRM Calibration Status**	Success	**DA Method**	PCDL.m
**Comment**			

**Sample Group**		**Info.**
**Acquisition SW**	6200 series TOF/6500 series	
**Version**	Q-TOF B.05.01 (B5125.2)	

User Spectra

Fragmentor Voltage	Collision Energy	Ionization Mode
135	0	ESI

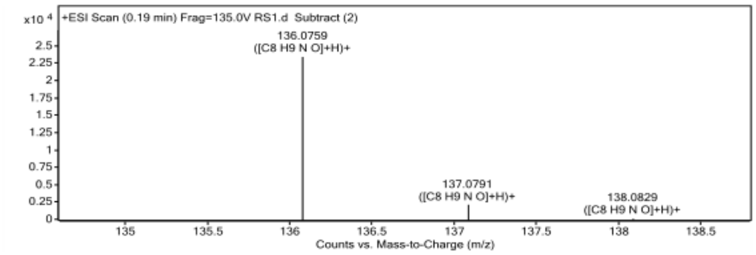

Peak List

m/z	z	Abund	Formula	Ion
136.0759	1	23525.47	C8 H9 N O	(M+H)+
158.0578	1	8511.72		
349.184	1	3729.81		
393.2096	1	3115.03		
437.2353	1	2878.5		
588.41	1	3202.58		
701.495	1	9609.03		
702.5003	1	4550.32		
814.5789	1	7417.28		
815.5824	1	4182.02		

Formula Calculator Element Limits

Element	Min	Max
C	3	60
H	0	120
O	0	30
N	0	3
S	0	3

Formula Calculator Results

Formula	CalculatedMass	CalculatedMz	Mz	Diff. (mDa)	Diff. (ppm)	DBE
C8 H9 N O	135.0684	136.0757	136.0759	-0.20	-1.47	5.0000

--- End Of Report ---

Figure S5.1 ^{1}H NMR spectrum of RS2 in CD_3OD

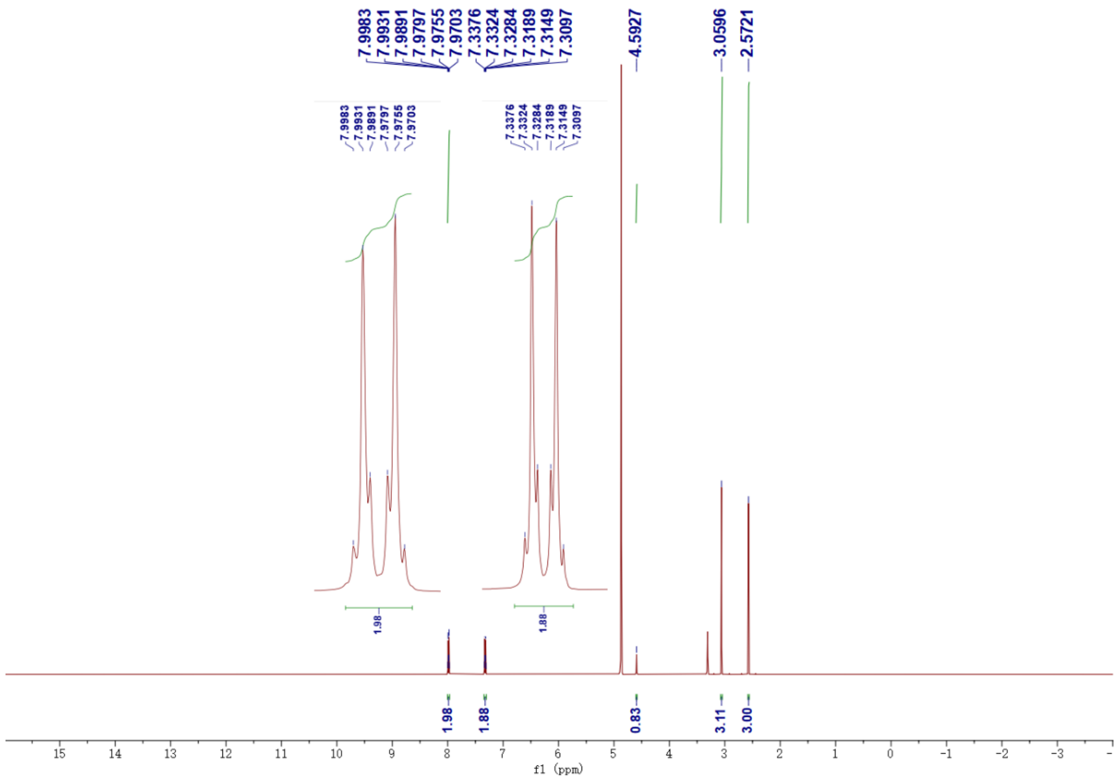

Figure S5.2 ^{13}C NMR spectrum of RS2 in CD_3OD

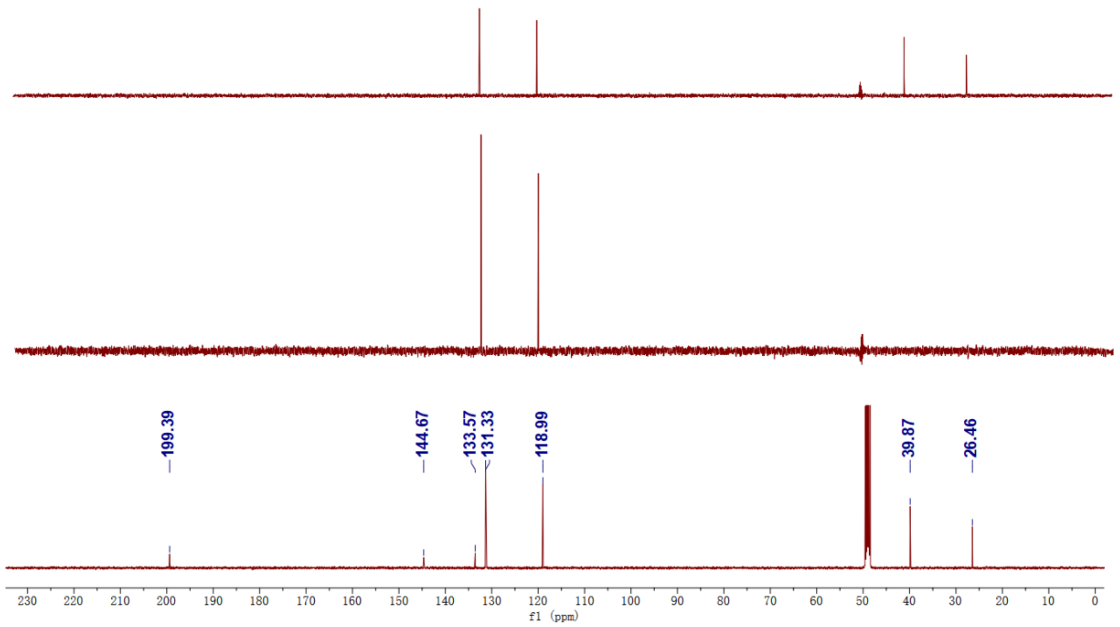

Figure S5.3 HRESIMS spectrum of RS2

Qualitative Analysis Report

**Data Filename**	RS2-.d	**Sample Name**	RS2
**Sample Type**	Sample	**Position**	P1-B3
**Instrument Name**	Instrument 1	**User Name**	
**Acq Method**	s-.m	**Acquired Time**	8/22/2022 4:49:48 PM
**IRM Calibration Status**	Success	**DA Method**	PCDL.m
**Comment**			

**Sample Group**		**Info.**
**Acquisition SW**	6200 series TOF/6500 series	
**Version**	Q-TOF B.05.01 (B5125.2)	

User Spectra

Fragmentor Voltage	Collision Energy	Ionization Mode
135	0	ESI

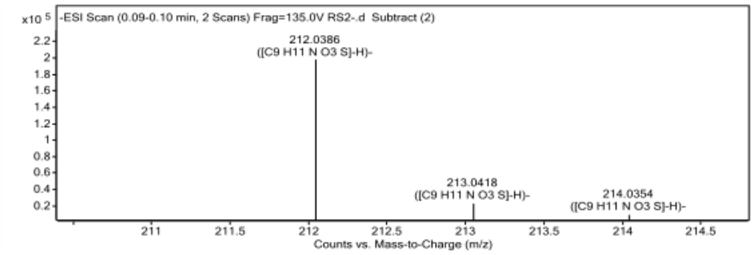

Peak List

m/z	z	Abund	Formula	Ion
133.0529	1	5192.46		
134.0605	1	6864.87		
212.0386	1	198340.78	C9 H11 N O3 S	(M-H)-
213.0418	1	24395.58	C9 H11 N O3 S	(M-H)-
214.0354	1	10774.88	C9 H11 N O3 S	(M-H)-
297.0161	1	5272.23		
348.0136	1	4083.97		
447.0662	1	11666.21		
471.2747	1	12397.93		
472.2783	1	3433.79		

Formula Calculator Element Limits

Element	Min	Max
C	3	60
H	0	120
O	0	30
N	0	3
S	0	3

Formula Calculator Results

Formula	CalculatedMass	CalculatedMz	Mz	Diff. (mDa)	Diff. (ppm)	DBE
C9 H11 N O3 S	213.0460	212.0387	212.0386	0.10	0.47	5.0000

--- End Of Report ---

Figure S6.1 ^{1}H NMR spectrum of RS3 in $CDCl_3$

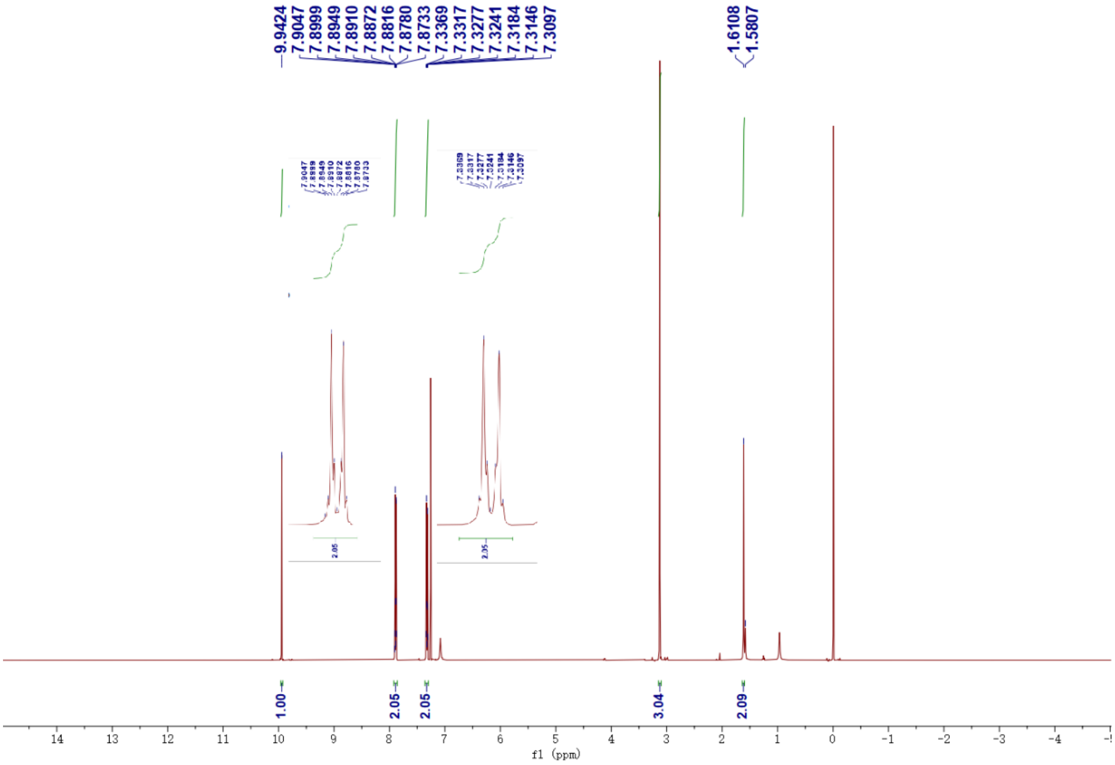

Figure S6.2 ^{13}C NMR spectrum of RS3in $CDCl_3$

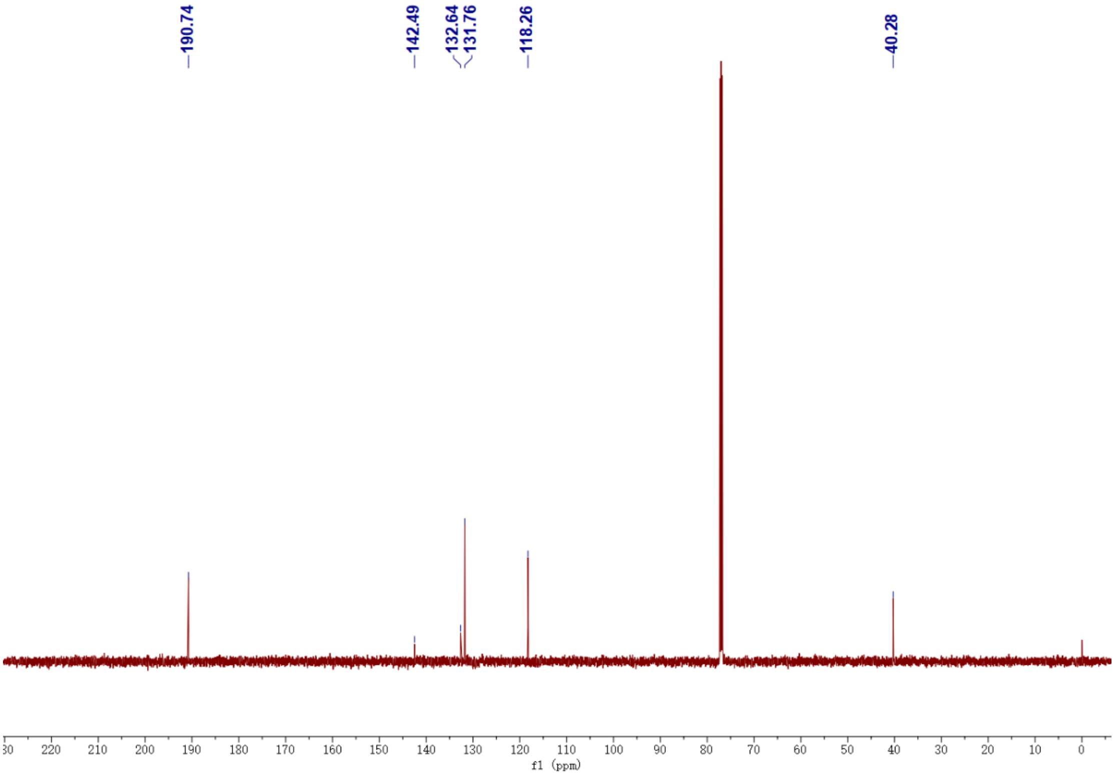

Figure S6.3 HRESIMS spectrum of RS3

Qualitative Analysis Report

**Data Filename**	RS3-.d	**Sample Name**	RS3
**Sample Type**	Sample	**Position**	P1-B4
**Instrument Name**	Instrument 1	**User Name**	
**Acq Method**	s-.m	**Acquired Time**	8/22/2022 4:50:46 PM
**IRM Calibration Status**	Success	**DA Method**	PCDL.m
**Comment**			

Sample Group **Info.**
Acquisition SW Version 6200 series TOF/6500 series Q-TOF B.05.01 (B5125.2)

User Spectra

Fragmentor Voltage	Collision Energy	Ionization Mode
135	0	ESI

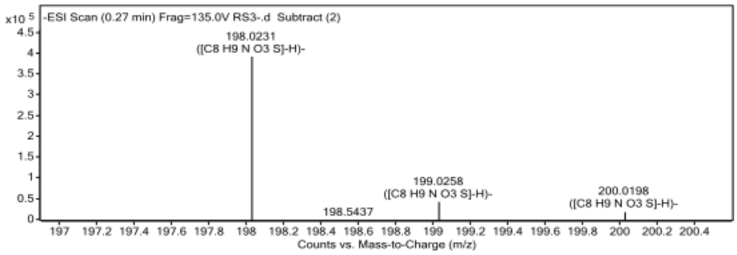

Peak List

m/z	z	Abund	Formula	Ion
120.043	1	13732.47		
121.0486	1	3846.79		
198.0231	1	392664.34	C8 H9 N O3 S	(M-H)-
199.0258	1	43927.92	C8 H9 N O3 S	(M-H)-
200.0198	1	20884.49	C8 H9 N O3 S	(M-H)-
283.0003	1	5716.74		
333.9976	1	5367.96		
419.0345	1	16222.55		
420.0386	1	3568.73		
471.2738	1	10529.69		

Formula Calculator Element Limits

Element	Min	Max
C	3	60
H	0	120
O	0	30
N	0	3
S	0	3

Formula Calculator Results

Formula	CalculatedMass	CalculatedMz	Mz	Diff. (mDa)	Diff. (ppm)	DBE
C8 H9 N O3 S	199.0303	198.0230	198.0231	-0.10	-0.50	5.0000

--- End Of Report ---
